# Shifts in diversification rates and host jump frequencies shaped the diversity of host range among *Sclerotiniaceae* fungal plant pathogens

**DOI:** 10.1111/mec.14523

**Published:** 2018-03-23

**Authors:** Olivier Navaud, Adelin Barbacci, Andrew Taylor, John P. Clarkson, Sylvain Raffaele

**Affiliations:** ^1^ LIPM, Université de Toulouse, INRA, CNRS Castanet‐Tolosan France; ^2^ Warwick Crop Centre School of Life Sciences University of Warwick Coventry UK

**Keywords:** angiosperms, coevolution, fungi, host parasite interactions

## Abstract

The range of hosts that a parasite can infect in nature is a trait determined by its own evolutionary history and that of its potential hosts. However, knowledge on host range diversity and evolution at the family level is often lacking. Here, we investigate host range variation and diversification trends within the *Sclerotiniaceae*, a family of Ascomycete fungi. Using a phylogenetic framework, we associate diversification rates, the frequency of host jump events and host range variation during the evolution of this family. Variations in diversification rate during the evolution of the *Sclerotiniaceae* define three major macro‐evolutionary regimes with contrasted proportions of species infecting a broad range of hosts. Host–parasite cophylogenetic analyses pointed towards parasite radiation on distant hosts long after host speciation (host jump or duplication events) as the dominant mode of association with plants in the *Sclerotiniaceae*. The intermediate macro‐evolutionary regime showed a low diversification rate, high frequency of duplication events and the highest proportion of broad host range species. Our findings suggest that the emergence of broad host range fungal pathogens results largely from host jumps, as previously reported for oomycete parasites, probably combined with low speciation rates. These results have important implications for our understanding of fungal parasites evolution and are of particular relevance for the durable management of disease epidemics.

## 1 INTRODUCTION

The host range of a parasite has a central influence on the emergence and spread of disease (Woolhouse & Gowtage‐Sequeria, 2005). There is a clear demarcation between specialist parasites that can only infect one or a few closely related host species, and generalists that can infect more than a hundred unrelated host species (Barrett, Kniskern, Bodenhausen, Zhang, & Bergelson, 2009; Woolhouse, Taylor, & Haydon, 2001). Host specialization, when lineages evolve to infect a narrower range of hosts than related lineages, is a frequent occurrence in living systems and can be driven by a parasite sharing habitat with only a limited number of potential hosts. There are also clear examples of parasite adaptations that restrict the use of co‐occurring potential hosts. For instance, the red rust fungal pathogen *Coleosporium ipomoeae* infects fewer *Ipomoea* species from its native community than when inoculated to non‐native communities, implying that evolution within local communities has narrowed pathogen host range (Chappell & Rausher, 2016). Some isolates of the rice blast fungus *Magnaporthe oryzae* are able to infect *Oryza sativa* japonica varieties but not indica varieties co‐occurring in the Yuanyang area of China, because their genome harbours numerous avirulence effector genes (Liao et al., 2016). It has been proposed that specialization results from trade‐offs between traits needed to infect a wide range of hosts (Futuyma & Moreno, 1988; Joshi & Thompson, 1995). It is frequently associated with the loss of traits that are not required to infect a particular host, such as the loss of lipid synthesis in parasitoid wasps (Visser et al., 2010), and the loss of secondary metabolism and carbohydrate active enzymes in powdery mildew fungal pathogens (Spanu et al., 2010). Specialization is sometimes considered as an evolutionary dead end (Moran, 1988), as gene losses are often irreversible and may lead specialist lineages “down a blind alley” that limits transitions back to generalism (Day, Hua, & Bromham, 2016; Haldane, 1951). There is nevertheless evidence for transitions from specialist to generalist parasitism (Hu et al., 2014; Johnson, Malenke, & Clayton, 2009). For instance in plant pathogens, *Pseudoperonospora cubensis* differs from other downy mildew oomycete pathogens in that it is able to infect a wide range of Cucurbits (Thines & Choi, 2015). For many parasite lineages however, knowledge on host range diversity and evolution at the macro‐evolutionary level is lacking.

*Sclerotiniaceae* is a family of Ascomycete fungi of the class Leotiomycetes which includes numerous plant parasites. Among the most studied are the grey mould pathogen *Botrytis cinerea*, considered to be one of the 10 most devastating plant pathogens (Dean et al., 2012), and the white and stem mould pathogen *Sclerotinia sclerotiorum*. Both are economically important pathogens in agriculture that infect hundreds of host plant species (Bolton, Thomma, & Nelson, 2006; Mbengue et al., 2016). The *Sclerotiniaceae* family also includes host specialist parasites such as *Ciborinia camelliae* that causes flower blight on *Camellia* (Denton‐Giles, Bradshaw, & Dijkwel, 2013), *Sclerotinia glacialis* that specifically infects *Ranunculus glacialis* (Graf & Schumacher, 1995), and *Monilinia oxycocci* causing the cottonball disease on cranberry (McManus, Best, & Voland, 1999). Other species from the *Sclerotiniaceae* have intermediate host range (tens of plant species) such as *Sclerotinia trifoliorum*,* S. subarctica* and *S. borealis* (Clarkson, Carter, & Coventry, 2010; Farr & Rossman, 2016). While *S. sclerotiorum* and *B. cinerea* are considered as typical necrotrophic pathogens, rapidly killing host cells to cause disease, the *Sclerotiniaceae* include species with diverse lifestyles. For instance, the poplar pathogen *Ciborinia whetzelii* and several *Myriosclerotinia* species are biotrophs that can live as endophytes (Andrew, Barua, Short, & Kohn, 2012; Schumacher & Kohn, 1985), while *Coprotinia minutula* is coprophilous (Elliott, 1967). How this remarkable diversity evolved remains elusive. To gain insights into this question, knowledge of phylogenetic relationships and host range diversity at the macro‐evolutionary level is needed. Specifically, ancestral state reconstruction can provide insights into how fungal host range has changed over time. The relationship between host range and diversification rates is an active area of research in insect ecology (Hamm & Fordyce, 2015; Hardy & Otto, 2014). For instance, a positive correlation was reported between species richness in the butterfly family *Nymphalidae* and the diversity of plants they feed upon (Janz, Nylin, & Wahlberg, 2006). However in contrast, some studies found a negative relationship between host‐plant breadth and diversification rate (Hardy & Otto, 2014). Transitions between feeding strategies generally appeared to be associated with shifts in insect diversification rates (Hardy & Otto, 2014; Janz & Nylin, 2008). Estimating fungal species divergence times in the *Sclerotiniaceae* will allow testing of whether biological diversification is related to host range variation.

Host range is a trait determined not only by the evolutionary history of a parasite, but also by that of its potential hosts (Poulin & Keeney, 2008). Accounting for host association patterns should therefore prove useful to understand host range evolution in the *Sclerotiniaceae*. The Leotiomycete class diverged less than 200 million years ago (Beimforde et al., 2014; Prieto & Wedin, 2013) and likely radiated with the diversification of flowering plants (Smith, Beaulieu, & Donoghue, 2010). Molecular phylogenetic studies distinguished *Myriosclerotinia*,* Sclerotinia* sensu stricto, *Botrytis* and *Botryotinia*, and *Monilini*a sensu stricto as monophyletic clades within the *Sclerotiniaceae* family (Holst‐Jensen, Vaage, & Schumacher, 1998; Holst‐Jensen, Vrålstad, & Schumacher, 2004). A phylogenetic analysis of *Monilinia* species suggested that cospeciation with host plants was the dominant pattern in this clade (Holst‐Jensen, Kohn, Jakobsen, & Schumacher, 1997). By contrast, there was no evidence for cospeciation with host plants in a phylogenetic analysis of *Botrytis* species (Staats, Van Baarlen, & Van Kan, 2005). *Botrytis* species were thus proposed to have evolved through host jumps to unrelated host plants followed by adaptation to their new hosts (Dong, Raffaele, & Kamoun, 2015; Staats et al., 2005). Host–parasite cophylogenetic analyses are required to test whether variations in the frequency of host jumps may have impacted on host range variation in the *Sclerotinaceae*.

In this study, we performed a phylogenetic analysis on 105 *Sclerotiniaceae* species to reveal multiple independent shifts and expansions of host range. We show that three macro‐evolutionary regimes with distinct diversification rates and dominant host association patterns have shaped the diversity of the *Sclerotiniaceae* and lead to contrasted proportions of broad host range species. Specifically, we highlight an increased emergence of broad host range parasites during the transition between macro‐evolutionary regimes dominated by distinct patterns of host–pathogen association. These results suggest that reduced diversification rates and high host jump frequency could associate with the emergence of generalist pathogens.

## 2 MATERIALS AND METHODS

### 2.1 Taxon and host range data selection

We used all 105 *Sclerotiniaceae* species for which at least *ITS* marker sequence data were available in the GenBank database. As outgroups we selected 56 *Rutstroemiaceae* species and 39 representative species of Leotiomycetes and Sordariomycetes for a total of 200 species. Host range data were obtained from (Boland & Hall, 1994; Melzer, Smith, & Boland, 1997), Index Fungorum (http://www.indexfungorum.org), the SMML fungus‐host distribution database (Farr & Rossman, 2016) and references therein. In total, we retrieved 7,101 fungus‐host association records that did not show strong geographic or crop/wild species bias (Figure S1). Fungal host range was not correlated to the number of database records (Figure S1c). Calibrated trees deciphering the relationships between the host families based on seven gene regions (18S rDNA, 26S rDNA, ITS, *matK*,* rbcL*,* atpB* and *trnL‐F*) were extracted from (Qian & Zhang, 2014) and updated with (Hedges, Marin, Suleski, Paymer, & Kumar, 2015). The tree of host families used for cophylogenetic analyses is provided as File S1.

### 2.2 Initial phylogenetic analysis

*ITS* sequences were aligned using mafft version 7 (Katoh & Standley, 2013). The alignment was manually adjusted to minimize possible homoplasic positions using Seaview 4 (Gouy, Guindon, & Gascuel, 2010). We retained gaps shorter than 41 positions present in a maximum of 39 sequences, with ungapped blocks being at least five characters long with a maximum of 5% ambiguous nucleotides per position. Unalignable and autapomorphic regions were excluded from the analysis, yielding an alignment with 797 informative sites (File S2). Maximum‐likelihood phylogeny was inferred with PhyML 3 (Guindon et al., 2010) with Smart Model Selection which permits an automatic substitution model selection supporting the general time‐reversible model with gamma distribution using six substitution rate categories (GTR+G6) model as the best fit. Statistical branch support was inferred with the SH‐like approximate likelihood ratio test (SH‐aLRT) (Anisimova, Gil, Dufayard, Dessimoz, & Gascuel, 2011) and bootstrap analysis with 100 replicates (Files 3 and 4). The topology of the tree was confirmed by three additional methods. First, we used a neighbour‐joining approach in FastME 2.0 (Lefort, Desper, & Gascuel, 2015) with the LogDet substitution model and tree refinement by subtree pruning and regrafting (File S5). Second, we used the parsimony ratchet approach implemented in the phangorn package in r (Schliep, 2011; File S6). Third, we used Bayesian analysis in MrBayes 3.2.6 (Ronquist et al., 2012) using the GTR substitution model with gamma‐distributed rate variation across sites and a proportion of invariable sites in 100 million MCMC generations, sampling parameters every 1,000, removing 10% of the tree files as burn‐in. The resulting trees, available in Dryad Digital Repository (https://doi.org/10.5061/dryad.7cs3g), were edited with Figtree (available at http://tree.bio.ed.ac.uk/software/figtree/).

### 2.3 Ancestral state reconstruction

To infer possible ancestral hosts, we used Reconstruct Ancestral State in Phylogenies 3.1 (RASP) (Yu, Harris, Blair, & He, 2015). We used the S‐DIVA (Statistical Dispersal‐Vicariance Analysis), S‐DEC (Statistical Dispersal–Extinction–Cladogenesis model), BBM (Bayesian Binary MCMC) and BayArea methods to verify congruence between the methods and assess the robustness of their output, as recommended by (Yu et al., 2015). We report the results of the S‐DIVA analysis which is considered the best adapted for host–parasite association analyses (Razo‐Mendivil & De Leon, 2011; Yu et al., 2015). To implement S‐DIVA, we delimited host groups as follows: Vitales (A), Asterids (B), Campanuliids (C), Commelinids (D), coprophilous (E), core Eudicots (F), Eudicots (G), Fabids (H), Polypodiidae (I), Lamiids (J), Magnoloidae (K), Malvids (L), Monocotyledones (M), Pinidae (N), allowing a maximum of four groups at each node. Among these groups, the latest divergence is that of Malvids and Fabids, estimated around 82.8 and 127.2 Mya (Clarke, Warnock, & Donoghue, 2011) and predates the emergence of *Sclerotiniaceae* estimated at ~69.7 Mya in this work; we therefore did not restrict associations in the ancestral reconstruction analysis. To account for incomplete sampling in this analysis, ancestral state reconstruction was computed for every plant group by the re‐rooting method (Yang, Kumar, & Nei, 1995) under an entity‐relationship (ER) model available in phytools (Revell, 2012) and derived from the ape library in R (Paradis, Claude, & Strimmer, 2004). This method, based on maximum likelihood, computed the probability to infect a given group of hosts for every nodes of the *Sclerotiniaceae* phylogeny. We considered plant groups as hosts when the probability of the ancestral state was >50%. For every plant group, up to 10% of terminal nodes were pruned randomly 100 times and the ancestral state reconstructed on pruned trees. For each plant group, we then extracted the variation in the age of the most ancestral inclusion into the *Sclerotiniaceae* host range (Figure S2). For all plant groups, this variation was not significantly different from 0, indicating that ancestral state reconstruction was robust to tree pruning.

### 2.4 Divergence dating analyses

We used a Bayesian approach to construct a chronogram with absolute times with the program beast 1.8.2. As no fossil is known in this fungal group, we use Sordariomycetes–Leotiomycetes divergence time to calibrate the tree (~300 Mya, Beimforde et al., 2014). Using our initial phylogeny as a constraint, the partition file was prepared with the BEAUti application of the beast package. Considering that our data set includes distantly related taxa, subsequent analyses were carried out using an uncorrelated relaxed clock model with lognormal distribution of rates. We used a uniform birth–death model with incomplete species sampling as prior on node age, following (Beimforde et al., 2014) to account for incomplete sampling in our phylogeny. Analyses were run three times for 100 million generations, sampling parameters every 5,000 generations, assessing convergence and sufficient chain mixing using Tracer 1.6 (Drummond, Suchard, Xie, & Rambaut, 2012). We removed 20% of trees as burn‐in, and the remaining trees were combined using LogCombiner (beast package), and summarized as maximum clade credibility (MCC) trees using TreeAnnotator within the beast package. The trees were edited in FigTree. The r packages *ape* (Paradis et al., 2004) and *phytools* (Revell, 2012) were used for subsequent analyses.

### 2.5 Diversification analysis

We used the section containing all 105 *Sclerotiniaceae* species of the chronogram with absolute times generated by beast to perform diversification analyses with three different methods. We first used Bayesian Analysis of Macroevolutionary Mixtures (BAMM) version 2.5 (Rabosky et al., 2013, 2014), an application designed to account for variation in evolutionary rates over time and among lineages. The priors were set using the setBAMMPriors command in the bammtools r‐package (Rabosky et al., 2014). We ran four parallel Markov chains for 5,000,000 generations and sampled for every 1,000 trees. The output and subsequent analyses were conducted with bammtools. We discarded the first 10% of the results and checked for convergence and the effective sample size (ESS) using the *coda* r‐package. Lineage‐through‐time plots with extant and extinct lineages were computed using the phytools package in r (Revell, 2012) with the drop.extinct=FALSE parameter. The exact number of *Sclerotiniaceae* species is currently unknown, and no intentional bias was introduced in data collection (Figure S1). To detect shifts in diversification rates taking into account incomplete sampling of the *Sclerotiniaceae* diversity and tree uncertainty, we used MEDUSA, a stepwise approach based upon the Akaike information criterion (AIC) (Alfaro et al., 2009; Drummond, Eastwood, Miotto, & Hughes, 2012). For this analysis, we used the birth and death (bd) model, allowed rate shifts both at stem and nodes, used the AIC as a statistical criterion with initial *r* = .05 and ε = 0.5. Sampling of the fungal diversity is typically estimated around 5% (Hawksworth & Luecking, 2017). We used random sampling rates between 5% and 100% for each individual species in 100 MEDUSA bootstrap replicates to estimate the impact of sampling richness. Next, we randomly pruned 1% to 10% of species from the tree in 100 MEDUSA bootstrap replicates to estimate the impact of tree completeness. To control for the impact of divergence time estimates on the diversification analysis, we altered all branching times in the tree randomly by −15% to +15% in 100 MEDUSA bootstrap replicates (Figure S3). Finally, we used rpanda which includes model‐free comparative methods for evolutionary analyses (Morlon et al., 2016). The model‐free approach in rpanda compares phylogenetic tree shapes based on spectral graph theory (Lewitus & Morlon, 2015). It constructs the modified Laplacian graph of a phylogenetic tree, a matrix with eigenvalues reflecting the connectivity of the tree. The density profile of eigenvalues (Figure S4a) provides information on the entire tree structure. The algorithm next used k‐medioids clustering to identify eight modalities in the phylogenetic tree of the *Sclerotiniaceae* (Figure S4b,c). A post hoc test comparing Bayesian information criterion (BIC) values for randomly bifurcated trees (BIC_random_) with that of the tree of the *Sclerotiniaceae* (BIC_Sclerotiniaceae_) supported the eight modalities (BIC_random_/BIC_Sclerotiniaceae_ = 10.34, well above the significance threshold of 4.0). We obtained significant BIC ratios with two or more modalities, supporting the existence of at least two macro‐evolutionary regimes in the *Sclerotiniaceae*.

### 2.6 Cophylogeny analysis

To detect potential codivergence patterns, we tested for cophylogeny between fungal species and host family trees with CoRe‐PA 0.5.1 (Merkle, Middendorf, & Wieseke, 2010), PACo (Balbuena, Míguez‐Lozano, & Blasco‐Costa, 2013) and Jane 4 (Conow, Fielder, Ovadia, & Libeskind‐Hadas, 2010) as these programs can accommodate pathogens with multiple hosts. The only three species which were not able to interact with an angiosperm host (saprotroph or only described on gymnosperms) were excluded from the analysis (namely: *Elliottinia kerneri*,* Coprotinia minutula* and *Stromatinia cryptomeriae*). We used a full set of 263 host–pathogen associations and a simplified set of 121 associations minimizing the number of host families involved to control for the impact of sampling bias (Table 1). We also performed the analyses independently for each macro‐evolutionary regime. To estimate cost parameters in CoRe‐PA, we ran a first cophylogeny reconstruction with cost values calculated automatically using the simplex method on 1,000 random cycles, with all host switches permitted, direct host switch and full host switch permitted (other parameters set to CoRe‐PA default). The best reconstruction had a quality score of 1.348 with total costs 37.076 and calculated costs of 0.0135 for cospeciation, 0.1446 for sorting, 0.2501 for duplication and 0.5918 for host switch. With these costs and parameters, we then computed reconstruction with 1,000 random associations to test for the robustness of the reconstructions. Next, we used 100,000 permutations of the host–pathogen association matrix in PACo to test for the overall congruence between host and pathogen trees. To test for the contribution of each association on the global fit, we performed taxon jackknifing. We used one‐tailed *z* tests to compare squared residuals distribution for each host–pathogen link with the median of squared residuals for the whole tree and infer likely co‐evolutionary and host shift links at *p* < .01. We used optimal costs calculated by CoRe‐PA to run cophylogeny reconstruction in Jane 4 Solve Mode with 50 generations and a population size of 1,000. Statistical tests were performed using 100 random tip mapping with 20 generations and population size of 100. For the analysis of each macro‐evolutionary regime in Jane 4, we used simplified host–pathogen association set to reduce the amount of Losses and Failure to Diverge predicted and facilitate comparisons with CoRe‐PA and PACo results. We visualized the interactions with TreeMap3 (Charleston & Robertson, 2002), using the untangling function to improve the layout.

**Table 1:** Results of the cophylogeny analyses under CoRe‐PA optimized cost settings using various methods and host association sets

		Event frequency (% of host associations)					
Method	Host associations^a^	Cospeciation	Sorting/Loss	Duplication	Host switch	FD	Cost/*p*‐val^b^
CoRe‐PA^c^	Full set (263)	15.82 ± 2.34	15.90 ± 4.80	34.35 ± 3.35	33.94 ± 3.49		38.21 ± 0.79
	Simplified (121)	9.63 ± 2.63	11.09 ± 4.97	40.20 ± 3.25	39.07 ± 4.00		40.44 ± 0.81
	G1 only (68)	15.75 ± 3.19	16.32 ± 7.35	37.78 ± 4.67	30.14 ± 5.23		13.95 ± 0.44
	G2 only (130)	17.03 ± 3.57	16.21 ± 7.58	33.16 ± 5.27	33.60 ± 5.63		14.57 ± 0.47
	G3 only (65)	11.78 ± 5.17	14.38 ± 8.69	32.86 ± 6.75	40.98 ± 6.87		8.95 ± 0.34
PACo^d^	Full set (263)	20.91	48.29		30.79		*p* < 1.0^−05^
	Simplified (121)	15.70	57.02		27.27		*p* < 1.0^−05^
	G1 only (68)	27.94	55.88		16.18		*p* = 2.0^−05^
	G2 only (130)	6.15	71.54		22.31		*p* = .00045
	G3 only (65)	0.00	66.15		33.85		*p* = .00607
Jane 4	Full set (263)	0.86	67.73	8.00	3.57	19.83	1,291 (*p* < .01)
	Simplified (121)	2.90	65.22	14.78	11.59	5.51	719.47 (*p* < .01)
	G1 simple (43)	1.39	41.67	25.00	26.39	5.56	206.98 (*p* < .01)
	G2 simple (54)	2.28	75.80	11.87	3.65	6.39	374.08 (*p* < .01)
	G3 simple (24)	9.68	25.81	19.35	41.94	3.23	105.41 (*p* = .13)

a Refers to the set of host association tested for cophylogeny: “Full set” corresponds to the complete list of all plant‐*Sclerotiniaceae* associations; “Simplified” corresponds to a reduced set covering the whole *Sclerotiniaceae* family; “G1,” “G2” and “G3” corresponds to associations involving *Sclerotiniaceae* species from macro‐evolutionary regime G1, G2 or G3 only.

b Values correspond to CoRe‐Pa total reconstruction costs, *p*‐value of the observed host–parasite association matrix m² in 100,000 permutations with PACo, or the *p*‐value of the observed reconstruction cost in 100 random tip mappings in Jane 4. FD, “failure to diverge,” corresponding to events when a host speciates and the parasite remains on both new host species.

c Standard deviation corresponds to frequencies calculated for 1,000 reconstruction with randomized host–parasite associations. Associations are classified as “cospeciation” when speciation of host and pathogen occurs simultaneously; “duplication” when pathogen speciation occurs independently of host speciation; “sorting or loss” when a pathogen remains associated with a single descendant host species after host speciation; and “host switch” when a pathogen changes host independently of speciation events.

d In PACo taxon jackknifing, associations that contributed significantly and positively to cophylogeny were classified as “cospeciation,” significantly and negatively as “host switch” and associations with no significant contribution to cophylogeny are classified as either sorting/loss or duplication.

## 3 RESULTS

### 3.1 Multiple independent shifts and expansions of host range in the evolution of the *Sclerotiniaceae*

**Figure 1:**
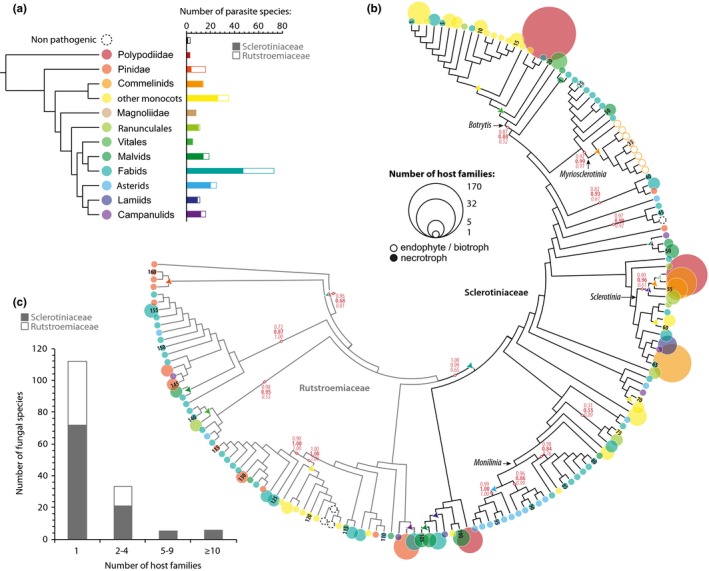
Multiple independent shifts and expansions of host range in the evolution of the *Sclerotiniaceae*. (a) Distribution of plant hosts of parasites from the *Sclerotiniaceae* and *Rutstroemiaceae* fungi. (b) Maximum‐likelihood *ITS* phylogeny of 105 *Sclerotiniaceae* and 56 Rutstroemiaceae species showing host range information and ancestral host reconstruction. Host range is shown as circles at the tips of branches, sized according to the number of host families and coloured as in (a) according to the earliest diverging plant group in host range. Numbers at the tips of branches refer to species listed in Table S1. Branch support indicated in light red for major clades corresponds to SH‐aLRT (regular), bootstrap (bold) and Bayesian posterior probabilities (italics). Reconstructed ancestral host is shown as triangles at intermediate nodes when a change compared to the previous node is predicted. Endophytes and biotrophic parasites are shown with empty circles. (c) Distribution of *Sclerotiniaceae* and *Rutstroemiaceae* species according to their number of host families [Colour figure can be viewed at http://wileyonlinelibrary.com]

To document the extant diversity in the *Sclerotiniaceae* fungi, we collected information on the host range of 105 species in this family. For comparison purposes, we also analysed the host range of 56 species from the *Rutstroemiaceae* family, a sister group of the *Sclerotiniaceae* (Figure 1a, Table S1). To reduce biases that may arise due to missing infection reports, we analysed host range at the family level instead of genus or species level. We found one species in the *Sclerotiniaceae* (*Coprotinia minutula*) and two species in the *Rutstroemiaceae* (*Rutstroemia cunicularia* and *Rutstroemia cuniculi*) reported as nonpathogenic to plants that are coprophilous (Elliott, 1967). Most species in the *Sclerotiniaceae* are reported necrotrophic pathogens, such as *Botrytis*,* Monilinia* and *Sclerotinia* species. Exceptions include *Ciborinia whetzelii* which was reported as an obligate biotroph of poplar (Andrew et al., 2012) and *Myriosclerotinia* species that specifically infect monocot families as facultative biotrophs or symptomless endophytes (Andrew et al., 2012). The most frequently parasitized plant group was Fabids (by 47 *Sclerotiniaceae* species and 26 *Rutstroemiaceae* species), a group of the Rosids (Eudicots) including notably cultivated plants from the Fabales (legumes) and Rosales (rose, strawberry, apple…) orders. Plants from the order Vitales (e.g., grapevine), from the Magnoliids and from the *Polypodiidae* (ferns) were colonized by *Sclerotiniaceae* but not *Rutstroemiaceae* fungi.

We next used the internal transcribed spacer (*ITS*) region of rDNA sequences to construct a phylogenetic tree of the 105 *Sclerotiniaceae* and 56 *Rutstroemiaceae* species (Figure 1b, Files S1–S7). This marker is the only one available for all species and is currently considered as the universal marker for taxonomic use (Schoch et al., 2012). Maximum‐likelihood, neighbour‐joining, parsimony and Bayesian analyses yielded convergent tree topologies. The tree topologies confirmed the familial classification recognized in previous phylogenies (Andrew et al., 2012; Holst‐Jensen et al., 1997, 1998), identifying a clearly supported *Botrytis* genus including 23 species; a *Myriosclerotinia* genus including eight species; a *Sclerotinia* genus sensu stricto including four *Sclerotinia*; and a *Monilinia* genus including 16 *Monilinia* species and three other species. *Botrytis calthae* and *Amphobotrys ricini* were the only two species with imprecise positions in maximum‐likelihood reconstructions, due to the *ITS* marker being insufficient to infer complete phylogenetic placement (Lorenzini & Zapparoli, 2016; Staats et al., 2005).

We used RASP (Yu et al., 2015) and phytools (Revell, 2012) to reconstruct the ancestral host range across the phylogeny (Figures 1b, S1 and S5). This identified Fabids as the most likely ancestral hosts of the *Sclerotiniaceae* family (relative probability of 100% in S‐DIVA and S‐DEC analyses). Random tree pruning in phytools indicated that this result is robust to sampling biases. Over 48% of the *Sclerotiniaceae* species are pathogens of host plants that evolved prior to the divergence of the Fabids, suggesting numerous host jumps in this family of parasites. Notably, a jump to Malvids and then to Monocots was identify at the base of the *Botrytis* genus (87% and 85% probability in S‐DIVA, respectively), a jump to Commelinids occurred in the *Myriosclerotinia* genus (91% probability in S‐DIVA), a jump to the Ranunculales occurred at the base of the *Sclerotinia* genus (76% probability in S‐DIVA); a jump to Asterids was found at the base of a major group of *Monilinia* (89% probability in S‐DIVA).

A total of 73 *Sclerotiniaceae* species (69.5%) and 41 (73.2%) *Rutstroemiaceae* infected a single host family (Figure 1c, Table S1). *Moellerodiscus lentus* was the only *Rutstroemiaceae* species infecting hosts from more than five plant families whereas eleven *Sclerotiniaceae* species (10.4%) exhibited this trait, including *Botrytis cinerea*,* Sclerotinia sclerotiorum*,* Sclerotinia minor* and *Grovesinia pyramidalis*, each of which colonizes plants from more than 30 families. Each of these species belongs to a clearly distinct phylogenetic group with a majority of species infecting a single host family. This may result from radiation following host jumps (Choi & Thines, 2015) and suggests that the ability to colonize a broad range of plant was acquired multiple times independently through the evolution of the *Sclerotiniaceae*.

### 3.2 Two major diversification rate shifts in the evolution of the *Sclerotiniaceae*

**Figure 2:**
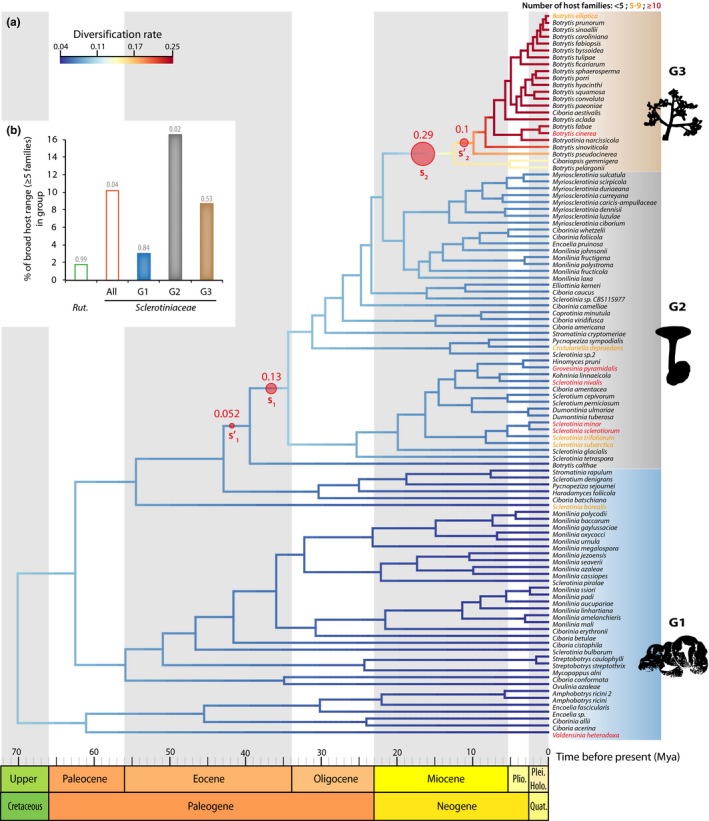
Two major diversification rate shifts in the evolution of the *Sclerotiniaceae*. (a) Dated *ITS*‐based species tree for the *Sclerotiniaceae* with diversification rate estimates. The divergence times correspond to the mean posterior estimate of their age in millions of years calculated with beast. Mean age is shown for selected nodes with bars showing 95% confidence interval of the highest posterior density (HPD). Branches of the tree are colour‐coded according to diversification rates determined with BAMM. Major rate shifts identified in BAMM are shown as red circles, noted *s*_1_, $s_{1}^{\prime }$, *s*_2_ and $s_{2}^{\prime }$ and labelled with the posterior distribution in the 95% credible set of macro‐evolutionary shift configurations. Species names are shown in black if host range includes less than five plant families, in yellow for five to nine plant families and in red for 10 or more plant families. Diversification rate shifts define three macro‐evolutionary regimes noted G1, G2 and G3 and boxed in blue, grey and brown, respectively. (b) Distribution of broad host range (five or more host families) parasites in *Rutstroemiaceae*,* Sclerotiniaceae* and under each macro‐evolutionary regime of the *Sclerotiniaceae*. *p*‐Values calculated by random permutations of host ranges along the tree are indicated above bars. Holo., Holocene; Mya, Million years ago; Plei., Pleistocene; Plio., Pliocene; Quat. Quaternary; Rut., *Rutstroemiaceae* [Colour figure can be viewed at http://wileyonlinelibrary.com]

To test for a relationship between host range variation and biological diversification in the *Sclerotiniaceae*, we estimated divergence times for the fungal family *Sclerotiniaceae* using the *ITS* marker in a Bayesian framework. A calibrated maximum clade credibility chronogram from these analyses is shown in Figure 2a (and Figure S6). This showed that *Sclerotiniaceae* fungi shared a most recent common ancestor between 33.9 and 103.5 million years ago (Mya), with a mean age of 69.7 Mya. The divergence of *Botrytis pseudocinerea*, estimated from the *ITS* data set, occurred ca. 3.35–17.8 Mya (mean age 9.8 Mya) and is similar to a previous estimate of 7–18 Mya (Walker et al., 2011).

**Figure 3:**
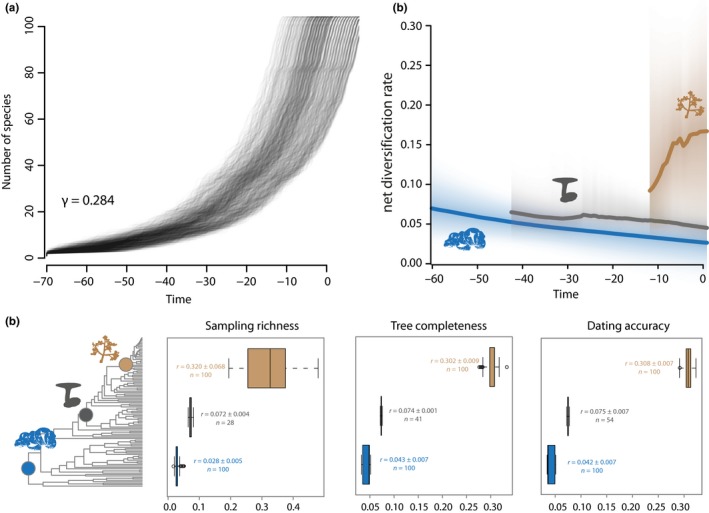
Robustness of diversification rate shifts identification in the *Sclerotiniaceae* phylogeny. (a) Lineage‐through‐time plots for the *Sclerotiniaceae* tree and 1,000 trees in which branching times were altered randomly by −15% to +15% to control for the sensitivity to divergence time estimates. Pybus γ for the *Sclerotiniaceae* tree is provided. (b) Frequency (*n*) of diversification rate shift detection and diversification rate estimates (*r*) in a 100 MEDUSA bootstrap replicates in which sampling richness, tree completeness and divergence times were randomly altered. Labels indicate average diversification rate estimates (*r*) for each macro‐evolutionary regime (blue for G1, grey for G2, brown for G3), with standard deviation of the mean for a 100 replicates. (c) Net diversification rates over time estimated by BAMM for each macro‐evolutionary regime (blue for G1, grey for G2, brown for G3) [Colour figure can be viewed at http://wileyonlinelibrary.com]

Using this time‐calibrated phylogeny, we calculated speciation and diversification rates using three different methods, to control for the limits of individual methods (Rabosky, Mitchell, & Chang, 2017). First, we used the BAMM framework, which implements a Metropolis Coupled Markov chain Monte Carlo method to calculate diversification rates along lineages (Rabosky, 2014) (Figures 2a and 7a–c). The analysis identified two significant rate shifts (*s*_1_ and *s*_2_, accounting for >30% of the posterior distribution in the 95% credible set of macro‐evolutionary shift configurations) each immediately adjacent to a minor rate shift ($s_{1}^{\prime }$ and $s_{2}^{\prime }$, respectively; ≤10% of the posterior distribution) within two different clades (Figure 2a). Each shift affects a specific clade and is not detected coincidentally across the whole phylogeny. The earliest shift occurred between 34.2 and 42.7 Mya. It resulted in an increase in instantaneous diversification rate from ~0.05 lineage/million year in *Monilinia* and related clades to ~0.08 lineage/million year in *Sclerotinia* and derived clades. The second shift occurred between 9.8 and 21.7 Mya. It resulted in a further increase in instantaneous diversification to >0.15 lineage/million year in *Botrytis* lineage. These two shifts defined three distinct macro‐evolutionary regimes in the *Sclerotiniaceae*. Second, to determine how speciation rates changed over time, we computed lineage‐through‐time plots for the *Sclerotiniaceae* tree and 1,000 trees in which we altered all branching times randomly by −15% to +15% to control for the sensitivity to divergence time estimates (Figure 3a). We observed an exponential trend of species accumulation consistent with a late burst of cladogenesis or early extinction (Pybus γ = 0.284). Third, we used MEDUSA (Alfaro et al., 2009; Drummond, Eastwood, et al., 2012) to detect shifts in diversification rate in the *Sclerotiniaceae* phylogeny. MEDUSA reported two shifts, corresponding to shifts *s*_1_ and $s_{2}^{\prime }$ identified in BAMM (Figure S3d). We used a bootstrap approach to test for the sensitivity of these shifts to sampling richness, tree completeness and dating accuracy (Figure 3b, Figure S3). Shift $s_{2}^{\prime }$ was detected in 100% of the bootstraps. Shift *s*_1_ was the second most frequent shift detected in our bootstrap analysis although it appeared sensitive to sampling richness in particular. Estimates of speciation rates in MEDUSA were robust to sampling richness, tree completeness and dating accuracy (*r* = .038 in G1, *r* = .074 in G2 and *r* = .31 in G3). These values were consistent with diversification rate estimated in BAMM for each regime (Figure 3c). Fourth, we used model‐free analysis of branching patterns in phylogenetic trees with R‐PANDA (Morlon et al., 2016). The spectrum of eigenvalues suggested eight modes of division in the phylogeny of the *Sclerotiniaceae* (Figure S4a,b). Comparison to randomly bifurcating trees suggested that these modalities are significant (BIC ratio 0.09). The modalities identified by rpanda k‐means clustering algorithm overlapped with the three macro‐evolutionary regimes identified previously (Figure S4c). Overall, these analyses converged towards the identification of three macro‐evolutionary regimes in the *Sclerotiniaceae*: species in regime G1 are early diverging and showed low diversification rates, and they encompassed notably *Monilinia*,* Encoelia* and some *Ciboria* species. Regime G2 had intermediate diversification rates and encompassed notably *Myriosclerotinia* species and most *Sclerotinia* species. Regime G3 corresponding to *Botrytis* genus was the most recently diverged and presented the highest diversification rates.

We compared the proportion of broad host range (≥5 plant families) fungal species that emerged in the *Rutstroemiaceae*, in the *Sclerotiniaceae*, and under each the three macro‐evolutionary regimes in the *Sclerotiniaceae* (Figure S2b). We randomly permuted host range values across the tree 10,000 times to estimate the *p*‐values of these proportions occurring by chance. We counted one (1.8%, *p* = .9959) broad host range species in the *Rutstroemiaceae* (*Moellerodiscus lentus*) and eleven (10.4%, *p* = .0359) in the *Sclerotiniaceae*. In the *Sclerotiniaceae*, regime G2 with intermediate diversification rates showed the highest proportion of broad host range species (16.2%, *p* = .0163), followed by regime G3 (8.7% of broad host range species, *p* = .537) and regime G1 (5.1% of broad host range species, *p* = .8407). These analyses suggest that the evolutionary history of regime G2 could have favoured the emergence of broad host range parasites in the *Sclerotiniaceae*.

### 3.3 Diversification rate shifts associate with variations in rates of cospeciation, duplication and host jump in the *Sclerotiniaceae*

**Figure 4:**
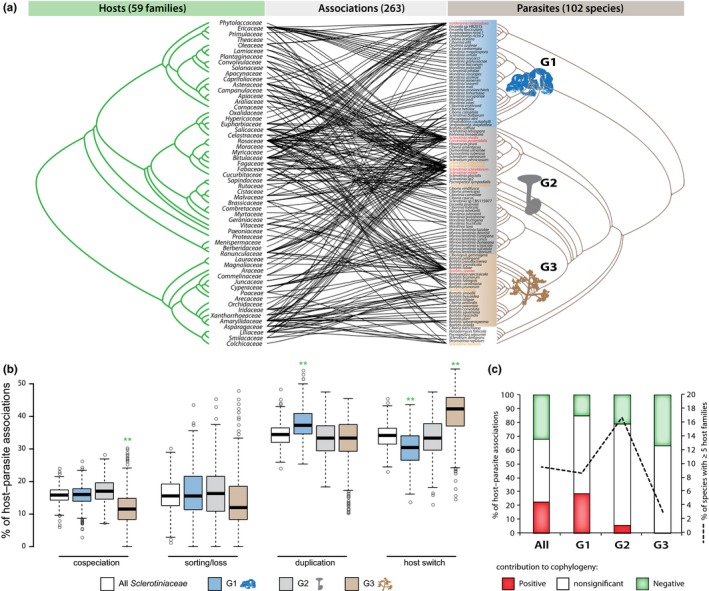
Diversification rate shifts associate with variations in rates of cospeciation, duplication and host switch in *Sclerotiniaceae* fungi. (a) Tanglegram depicting the associations between 102 *Sclerotiniaceae* species and 59 plant families. The three macro‐evolutionary regimes are indicated by coloured boxes on the *Sclerotiniaceae* tree. Fungal species labels are colour‐coded as in Figure 2. (b) Proportion of cospeciation, sorting/loss, duplication and host switches in host–*Sclerotiniaceae* associations as predicted by CoRe‐PA in 1,000 cophylogeny reconstructions. ** indicate large effect size in a macro‐evolutionary compared to the complete set of associations as assessed by Cohen's d test. (c) Proportion of host–*Sclerotiniaceae* associations contributing significantly and positively (likely cospeciation), non significantly and significantly and negatively (likely host switch) to cophylogeny in PACo analysis. The black dotted line indicates the percentage of broad host range species (five or more host families) in each group [Colour figure can be viewed at http://wileyonlinelibrary.com]

Within the last 50 Ma, the world has experienced an overall decrease in mean temperatures but with important fluctuations that dramatically modified the global distribution of land plants (Donoghue & Edwards, 2014; Nürk, Uribe‐Convers, Gehrke, Tank, & Blattner, 2015; Zachos, Pagani, Sloan, Thomas, & Billups, 2001). We hypothesized that modifications in the distribution of plants could have an impact on host association patterns in *Sclerotiniaceae* parasites. To test this hypothesis, we performed host–parasite cophylogeny reconstructions using CoRe‐PA (Merkle et al., 2010), PACo (Balbuena et al., 2013) and Jane 4 (Conow et al., 2010) (Table 1). We found that hosts of *Sclerotiniaceae* fungi include ~1,800 plant species. To consider this overall host diversity in a cophylogenetic analysis, we used a tree including 56 plant families, a tree of 102 *Sclerotiniaceae* parasitic species, and 263 host–parasite interactions (File S3). The resulting tanglegram (Figure 4a) highlighted clear parasite duplications notably on *Ericaceae* and *Rosaceae* in the G1 group of the *Sclerotiniaceae* (*Monilinia* species). There was no obvious topological congruence between the plant tree and groups G2 and G3 of the *Sclerotiniaceae*. To take into account sampling bias, we performed cophylogeny reconstructions using the full set of 263 host–parasite associations and a simplified set of 121 associations. Reconstructions were also performed independently on each of the three macro‐evolutionary regimes. CoRe‐PA classifies host–pathogen associations into (i) cospeciation, when speciation of host and pathogen occurs simultaneously, (ii) duplication, when pathogen speciation occurs independently of host speciation, (iii) sorting or loss, when a pathogen remains associated with a single descendant host species after host speciation, and (iv) host switch (also designated as host jump) when a pathogen changes host independently of speciation events (Merkle et al., 2010). The CoRe‐PA reconstructions indicated that duplications and host switch each represented ~34% of host associations from the full set of host–*Sclerotiniaceae* associations. Cospeciation and sorting each represented ~16% of host associations. The analysis of each macro‐evolutionary regime indicated that G1 is characterized by high duplication and low host jump frequencies and G3 by low cospeciation and sorting frequencies and high host jump frequencies (Figure 4b). We next used Procrustean superimposition in PACo (Balbuena et al., 2013) to identify host–pathogen associations that contribute significantly to cophylogeny between *Sclerotiniaceae* and host plant families. The PACo analysis suggested that overall, the *Sclerotiniaceae* lineages are not randomly associated with their host families (*p* < .01). The PACo analysis also includes taxon jackknifing to test for the relative contribution of each host–pathogen association in the cophylogeny pattern. In the full set of host–*Sclerotiniaceae* associations, ~21% contributed positively and significantly to cophylogeny, likely representing cospeciation events, while ~31% contributed negatively and significantly to cophylogeny, therefore likely representing host jump events (Table 1). Taxon jackknifing on individual macro‐evolutionary regime revealed a high frequency of associations with positive contribution to cophylogeny (likely cospeciation) in G1, and a high frequency of associations with negative contribution to cophylogeny (likely host switch) in G3, in good agreement with the CoRe‐PA analysis. In addition to the types of host–parasite association mentioned before, Jane 4 identifies “failure to diverge” associations, corresponding to events when a host speciates and the parasite remains on both new host species (Conow et al., 2010). Jane 4 analysis for the whole *Sclerotiniaceae* family (simplified set of associations) identifies losses as the dominant form of host association (~65%), followed by duplications (~15%) and host jumps (~12%) while failure to diverge (~5.5%) and cospeciation (~3%) was rare event (Table 1). Consistent with CoRe‐PA and PACo analyses, Jane 4 found the highest rate of host jumps in regime G3 (~42%). Unlike previous analyses, Jane 4 found the highest rate of cospeciation in G3 (~9.7%). G1 was characterized by a high duplication rate, while G2 was characterized by a high rate of losses and failure to diverge events but the lowest host jump rate. Overall, our cophylogeny analyses converged towards the conclusion that cospeciations represent a minor proportion of host associations in the *Sclerotiniaceae* and that the proportion of host jumps varied markedly between the three macro‐evolutionary regimes, with G3 showing the highest host jump rate (between 33% and 42%).

## 4 DISCUSSION

Our analyses lead to a model in which the extant diversity of *Sclerotiniaceae* fungi is the result of three macro‐evolutionary regimes characterized by distinct diversification rates and host association patterns. Patterns of cophylogeny decreased from regime G1 to G3, while the frequency of inferred host jump events increased from G1 to G3. Regime G2, which includes the highest proportion of broad host range parasites, showed a frequency of host jumps intermediate between G1 and G3. Our cophylogeny analyses are consistent with the view that long‐term plant‐pathogen cospeciation is rare (Devienne et al., 2013). The decrease in host‐pathogen cophylogeny signal from G1 to G3 regime (Figure 4) could indicate more frequent true cospeciation events in early diverging *Sclerotiniaceae* species or could result from host jumps being restricted to closely related hosts for G1 species whereas G2 and G3 species progressively gained the ability to jump to more divergent hosts (Devienne, Giraud, & Shykoff, 2007; Devienne et al., 2013). Low diversification rates in regimes G1 and G2 may have resulted from increased extinction rates. It is conceivable that a high extinction rate of specialist parasites during regime G2 is responsible for the reduced clade diversification rates, while increasing the apparent frequency of emergence of generalist species. This phenomenon, consistent with the view of specialization as “an evolutionary dead end” (leading to a reduced capacity to diversify), is notably supported in *Tachinidae* parasitic flies and hawkmoths pollinating *Ruellia* plants (Day et al., 2016). An analysis of *Papilionoidea* and *Heliconii* revealed lower rates of diversification for butterfly species feeding on a broad range of plants compared to specialist butterflies (Day et al., 2016; Hardy & Otto, 2014). Consistently, theoretical models of sympatric speciation predict that competition for a narrow range of resources (specialization) to be a strong driver of speciation (Dieckmann & Doebeli, 1999). Similar to plant‐feeding insects, the diversity of fungal and oomycete pathogens is considered largely driven by host jumps rather than host specialization that may follow (Choi & Thines, 2015; Hardy & Otto, 2014). Low diversification rates in G1 and G2 may also result from a strong increase in diversification rate during the transition from regime G2 to G3. Our ancestral state reconstruction analysis inferred a jump to monocots at the base of regime G3 (Figure 1). A recent study on *Hesperiidae* butterflies reported a strong increase in diversification coincident with a switch from dicot‐feeders to monocot‐feeders (Sahoo, Warren, Collins, & Kodandaramaiah, 2017). As postulated for *Hesperiidae*, the emergence of open grasslands and global temperature decrease may have affected diversification in the *Sclerotiniaceae*.

Competition for resources is likely to lead to speciation if host range expansion is costly (Ackermann, Doebeli, & Gomulkiewicz, 2004). Notably, if different host families are scattered in space and phylogenetically related hosts are clustered, the cost of host range expansion is expected to increase, due to higher costs for dispersal or trade‐offs with other traits (Ackermann et al., 2004). In agreement with this theory, genomic signatures associated with metabolic cost optimization were stronger in generalist than specialist fungal parasites (Badet et al., 2017). Strong climate oscillations during the Cenozoic Era, leading to population isolation through range fragmentations and dispersal events, likely contributed to the radiation of plant lineages (Nyman, Linder, Peña, Malm, & Wahlberg, 2012). For instance, major events in the diversification of *Brassicaceae* plant family coincide with glaciations and arid conditions of the Eocene–Oligocene and the Oligocene–Miocene transitions (Hohmann, Wolf, Lysak, & Koch, 2015). In addition, the mid‐Miocene (20–10 Mya) corresponds to the emergence of the first open grassland habitats in northern Eurasia (Strömberg, 2011). The fragmentation and diversification of plant host populations may have increased the cost for host range expansion in fungal pathogens. A jump to monocots at the base of *Sclerotiniaceae* regime G3 ~10 Mya may have favoured the conquest of grassland habitats and the rapid diversification of specialist species over the emergence of generalists in this group.

Similar to herbivore diet breadth (Forister et al., 2015), host range in the *Sclerotiniaceae* shows a continuous distribution from specialists to broad host range generalists, with the majority of species being specialists. Mathematical analyses and studies of the host range of insect herbivores suggest that host range expansion could involve the emergence of a “pre‐adaptation” followed by the colonization of new hosts (Janz & Nylin, 2008). The existence of such pre‐existing enabler traits has been proposed as a facilitator for several shifts in plant species distribution (Donoghue & Edwards, 2014). Notably, the pre‐existence of a symbiotic signalling pathway in algae is thought to have facilitated the association of land plants with symbiotic fungi (Delaux et al., 2015). In the plant genus *Hypericum*, increased diversification rates in the Miocene Epoch were likely facilitated by adaptation to colder climates (Nürk et al., 2015). In the bacterial pathogen *Serratia marcescens* and in yeast, adaptation to temperature change was associated with improved tolerance to other stresses (Caspeta & Nielsen, 2015; Ketola et al., 2013). Analysis of the complete predicted proteomes of *S. borealis*,* S. sclerotiorum* and *Botrytis cinerea* has revealed protein signatures often associated with cold adaptation in the secreted protein of all three species (Badet, Peyraud, & Raffaele, 2015). Cold tolerance might have been a pre‐adaptation that facilitated the emergence of generalist parasites under low diversification rates (regime G2) and a rapid diversification following host jumps on fragmented host populations (regime G3). Indeed, *Sclerotinia borealis*,* S. glacialis*,* S. subarctica* and *S. nivalis*, that diverged during regime G2, are largely restricted to hemiboreal climates (circumboreal region) and can have a lower optimal growth temperature than their sister species (Hoshino, Terami, Tkachenko, Tojo, & Matsumoto, 2010; Saito, 1997).

These findings suggest that global climate instability and host diversification in the Cenozoic might have impacted on the diversity of fungal parasites within the *Sclerotiniaceae*. This effect could have been direct, through the emergence of cold adaptation as an enabling trait, or indirect through changes in host population structures and host–parasite association patterns. Knowledge on the dynamics of pathogen evolution increases the understanding of the complex interplay between host, pathogen and environment governing the dynamics of disease epidemics. These evolutionary principles are useful for the design of disease management strategies (Vander Wal et al., 2014) and provide new insights into the factors that influenced the diversity of extant fungal parasite species.

## DATA ACCESSIBILITY

- Species identifiers: GenBank accessions provided in Table S1
- DNA sequences and alignments: provided as File S2
- Phylogenetic trees: provided as Files S1, S3, S4 and S5
- Host range data, DNA sequences and alignments and Phylogenetic trees are available from the Dryad Digital Repository: https://doi.org/10.5061/dryad.7cs3g

## AUTHOR CONTRIBUTIONS

O.N. collected data, performed analyses, wrote the original draft and revised the manuscript; A.B. performed analyses, wrote and revised the manuscript; A.T. collected data and revised the manuscript; J.P.C. involved in funding acquisition, data collection, revision of manuscript; S.R. involved in supervision, funding acquisition, project administration, writing original draft and revision of manuscript.

## References

[mec14523-bib-0001] Ackermann, M. , Doebeli, M. , & Gomulkiewicz, R. (2004). Evolution of niche width and adaptive diversification. Evolution, 58, 2599–2612. https://doi.org/10.1111/j.0014-3820.2004.tb01614.x 15696740 10.1111/j.0014-3820.2004.tb01614.x

[mec14523-bib-0002] Alfaro, M. E. , Santini, F. , Brock, C. , Alamillo, H. , Dornburg, A. , Rabosky, D. L. , … Harmon, L. J. (2009). Nine exceptional radiations plus high turnover explain species diversity in jawed vertebrates. Proceedings of the National Academy of Sciences of the United States of America, 106, 13410–13414. https://doi.org/10.1073/pnas.0811087106 19633192 10.1073/pnas.0811087106 PMC2715324

[mec14523-bib-0003] Andrew, M. , Barua, R. , Short, S. M. , & Kohn, L. M. (2012). Evidence for a common toolbox based on necrotrophy in a fungal lineage spanning necrotrophs, biotrophs, endophytes, host generalists and specialists. PLoS One, 7, e29943 https://doi.org/10.1371/journal.pone.0029943 22253834 10.1371/journal.pone.0029943 PMC3256194

[mec14523-bib-0004] Anisimova, M. , Gil, M. , Dufayard, J.‐F. , Dessimoz, C. , & Gascuel, O. (2011). Survey of branch support methods demonstrates accuracy, power, and robustness of fast likelihood‐based approximation schemes. Systematic biology, 60(5), 685–699. https://doi.org/10.1093/sysbio/syr041 21540409 10.1093/sysbio/syr041 PMC3158332

[mec14523-bib-0005] Badet, T. , Peyraud, R. , Mbengue, M. , Navaud, O. , Derbyshire, M. , Oliver, R. P. , … Raffaele, S. (2017). Codon optimization underpins generalist parasitism in fungi. eLife, 6, e22472. 28157073 10.7554/eLife.22472 PMC5315462

[mec14523-bib-0006] Badet, T. , Peyraud, R. , & Raffaele, S. (2015). Common protein sequence signatures associate with *Sclerotinia borealis* lifestyle and secretion in fungal pathogens of the *Sclerotiniaceae* . Frontiers in Plant Science, 6, 776. 26442085 10.3389/fpls.2015.00776 PMC4585107

[mec14523-bib-0007] Balbuena, J. A. , Míguez‐Lozano, R. , & Blasco‐Costa, I. (2013). PACo: A novel procrustes application to cophylogenetic analysis. PLoS One, 8, e61048 https://doi.org/10.1371/journal.pone.0061048 23580325 10.1371/journal.pone.0061048 PMC3620278

[mec14523-bib-0008] Barrett, L. G. , Kniskern, J. M. , Bodenhausen, N. , Zhang, W. , & Bergelson, J. (2009). Continua of specificity and virulence in plant host–pathogen interactions: Causes and consequences. New Phytologist, 183, 513–529. https://doi.org/10.1111/j.1469-8137.2009.02927.x 19563451 10.1111/j.1469-8137.2009.02927.x

[mec14523-bib-0009] Beimforde, C. , Feldberg, K. , Nylinder, S. , Rikkinen, J. , Tuovila, H. , Dörfelt, H. , … Schmidt, A. R. (2014). Estimating the Phanerozoic history of the Ascomycota lineages: Combining fossil and molecular data. Molecular phylogenetics and evolution, 78, 386–398. https://doi.org/10.1016/j.ympev.2014.04.024 24792086 10.1016/j.ympev.2014.04.024

[mec14523-bib-0010] Boland, G. , & Hall, R. (1994). Index of plant hosts of *Sclerotinia sclerotiorum* . Canadian Journal of Plant Pathology, 16, 93–108. https://doi.org/10.1080/07060669409500766

[mec14523-bib-0011] Bolton, M. D. , Thomma, B. P. H. J. , & Nelson, B. D. (2006). *Sclerotinia sclerotiorum* (Lib.) de Bary: Biology and molecular traits of a cosmopolitan pathogen. Molecular Plant Pathology, 7, 1–16. https://doi.org/10.1111/j.1364-3703.2005.00316.x 20507424 10.1111/j.1364-3703.2005.00316.x

[mec14523-bib-0012] Caspeta, L. , & Nielsen, J. (2015). Thermotolerant yeast strains adapted by laboratory evolution show trade‐off at ancestral temperatures and preadaptation to other stresses. MBio, 6, e00431‐15 https://doi.org/10.1128/mBio.00431-15 26199325 10.1128/mBio.00431-15 PMC4513085

[mec14523-bib-0013] Chappell, T. M. , & Rausher, M. D. (2016). Evolution of host range in *Coleosporium ipomoeae*, a plant pathogen with multiple hosts. Proceedings of the National Academy of Sciences of the United States of America, 113, 5346–5351. https://doi.org/10.1073/pnas.1522997113 27114547 10.1073/pnas.1522997113 PMC4868424

[mec14523-bib-0014] Charleston, M. , & Robertson, D. (2002). Preferential host switching by primate lentiviruses can account for phylogenetic similarity with the primate phylogeny. Systematic biology, 51, 528–535. https://doi.org/10.1080/10635150290069940 12079649 10.1080/10635150290069940

[mec14523-bib-0015] Choi, Y.‐J. , & Thines, M. (2015). Host jumps and radiation, not co‐divergence drives diversification of obligate pathogens. A case study in downy mildews and Asteraceae. PLoS One, 10, e0133655 https://doi.org/10.1371/journal.pone.0133655 26230508 10.1371/journal.pone.0133655 PMC4521919

[mec14523-bib-0016] Clarke, J. T. , Warnock, R. , & Donoghue, P. C. (2011). Establishing a time‐scale for plant evolution. New Phytologist, 192, 266–301. https://doi.org/10.1111/j.1469-8137.2011.03794.x 21729086 10.1111/j.1469-8137.2011.03794.x

[mec14523-bib-0017] Clarkson, J. P. , Carter, H. , & Coventry, E. (2010). First report of *Sclerotinia subarctica* nom. prov. (*Sclerotinia* species 1) in the UK on *Ranunculus acris* . Plant pathology, 59, 1173 https://doi.org/10.1111/j.1365-3059.2010.02271.x

[mec14523-bib-0018] Conow, C. , Fielder, D. , Ovadia, Y. , & Libeskind‐Hadas, R. (2010). Jane: A new tool for the cophylogeny reconstruction problem. Algorithms for Molecular Biology, 5, 16 https://doi.org/10.1186/1748-7188-5-16 20181081 10.1186/1748-7188-5-16 PMC2830923

[mec14523-bib-0019] Day, E. H. , Hua, X. , & Bromham, L. (2016). Is specialization an evolutionary dead end? Testing for differences in speciation, extinction and trait transition rates across diverse phylogenies of specialists and generalists. Journal of evolutionary biology, 29, 1257–1267. https://doi.org/10.1111/jeb.12867 26998589 10.1111/jeb.12867

[mec14523-bib-0020] Dean, R. , JaL, Van Kan , Pretorius, Z. A. , Hammond‐Kosack, K. E. , Di Pietro, A. , Spanu, P. D. , … Foster, G. D. (2012). The Top 10 fungal pathogens in molecular plant pathology. Molecular Plant Pathology, 13, 414–430. https://doi.org/10.1111/j.1364-3703.2011.00783.x 22471698 10.1111/j.1364-3703.2011.00783.x PMC6638784

[mec14523-bib-0021] Delaux, P.‐M. , Radhakrishnan, G. V. , Jayaraman, D. , Cheema, J. , Malbreil, M. , Volkening, J. D. , … Ané, J. M. (2015). Algal ancestor of land plants was preadapted for symbiosis. Proceedings of the National Academy of Sciences of the United States of America, 112, 13390–13395. https://doi.org/10.1073/pnas.1515426112 26438870 10.1073/pnas.1515426112 PMC4629359

[mec14523-bib-0022] Denton‐Giles, M. , Bradshaw, R. E. , & Dijkwel, P. P. (2013). *Ciborinia camelliae* (Sclerotiniaceae) induces variable plant resistance responses in selected species of Camellia. Phytopathology, 103, 725–732. https://doi.org/10.1094/PHYTO-11-12-0289-R 23406433 10.1094/PHYTO-11-12-0289-R

[mec14523-bib-0023] Devienne, D. , Giraud, T. , & Shykoff, J. (2007). When can host shifts produce congruent host and parasite phylogenies? A simulation approach. Journal of evolutionary biology, 20, 1428–1438. https://doi.org/10.1111/j.1420-9101.2007.01340.x 17584237 10.1111/j.1420-9101.2007.01340.x

[mec14523-bib-0024] Devienne, D. , Refrégier, G. , López‐Villavicencio, M. , Tellier, A. , Hood, M. , & Giraud, T. (2013). Cospeciation vs host‐shift speciation: Methods for testing, evidence from natural associations and relation to coevolution. New Phytologist, 198, 347–385. https://doi.org/10.1111/nph.12150 23437795 10.1111/nph.12150

[mec14523-bib-0025] Dieckmann, U. , & Doebeli, M. (1999). On the origin of species by sympatric speciation. Nature, 400, 354–357. https://doi.org/10.1038/22521 10432112 10.1038/22521

[mec14523-bib-0026] Dong, S. , Raffaele, S. , & Kamoun, S. (2015). The two‐speed genomes of filamentous pathogens: Waltz with plants. Current Opinion in Genetics & Development, 35, 57–65. https://doi.org/10.1016/j.gde.2015.09.001 26451981 10.1016/j.gde.2015.09.001

[mec14523-bib-0027] Donoghue, M. J. , & Edwards, E. J. (2014). Biome shifts and niche evolution in plants. Annual Review of Ecology, Evolution, and Systematics, 45, 547–572. https://doi.org/10.1146/annurev-ecolsys-120213-091905

[mec14523-bib-0028] Drummond, C. S. , Eastwood, R. J. , Miotto, S. T. , & Hughes, C. E. (2012). Multiple continental radiations and correlates of diversification in Lupinus (Leguminosae): Testing for key innovation with incomplete taxon sampling. Systematic biology, 61, 443–460. https://doi.org/10.1093/sysbio/syr126 22228799 10.1093/sysbio/syr126 PMC3329764

[mec14523-bib-0029] Drummond, A. J. , Suchard, M. A. , Xie, D. , & Rambaut, A. (2012). Bayesian phylogenetics with BEAUti and the beast 1.7. Molecular biology and Evolution, 29, 1969–1973. https://doi.org/10.1093/molbev/mss075 22367748 10.1093/molbev/mss075 PMC3408070

[mec14523-bib-0030] Elliott, M. E. (1967). Rutstroemia cuniculi, a coprophilous species of the *Sclerotiniaceae* . Canadian Journal of Botany, 45, 521–524. https://doi.org/10.1139/b67-053

[mec14523-bib-0031] Farr, D. F. , & Rossman, A. Y. (2016). Fungal Databases, Systematic Mycology and Microbiology Laboratory, ARS, USDA.

[mec14523-bib-0032] Forister, M. L. , Novotny, V. , Panorska, A. K. , Baje, L. , Basset, Y. , Butterill, P. T. , … Dyer, L. A. (2015). The global distribution of diet breadth in insect herbivores. Proceedings of the National Academy of Sciences of the United States of America, 112, 442–447. https://doi.org/10.1073/pnas.1423042112 25548168 10.1073/pnas.1423042112 PMC4299246

[mec14523-bib-0033] Futuyma, D. J. , & Moreno, G. (1988). The evolution of ecological specialization. Annual Review of Ecology and Systematics, 19(1), 207–233. https://doi.org/10.1146/annurev.es.19.110188.001231

[mec14523-bib-0034] Gouy, M. , Guindon, S. , & Gascuel, O. (2010). SeaView version 4: A multiplatform graphical user interface for sequence alignment and phylogenetic tree building. Molecular biology and Evolution, 27, 221–224. https://doi.org/10.1093/molbev/msp259 19854763 10.1093/molbev/msp259

[mec14523-bib-0035] Graf, F. , & Schumacher, T. (1995). Sclerotinia glacialis sp. nov., from the alpine zone of Switzerland. Mycological Research, 99, 113–117. https://doi.org/10.1016/S0953-7562(09)80324-9

[mec14523-bib-0036] Guindon, S. , Dufayard, J.‐F. , Lefort, V. , Anisimova, M. , Hordijk, W. , & Gascuel, O. (2010). New algorithms and methods to estimate maximum‐likelihood phylogenies: Assessing the performance of PhyML 3.0. Systematic biology, 59, 307–321. https://doi.org/10.1093/sysbio/syq010 20525638 10.1093/sysbio/syq010

[mec14523-bib-0037] Haldane, J. B. S. (1951). Everything has a history. London, UK: Routledge.

[mec14523-bib-0038] Hamm, C. A. , & Fordyce, J. A. (2015). Patterns of host plant utilization and diversification in the brush‐footed butterflies. Evolution, 69, 589–601. https://doi.org/10.1111/evo.12593 25546268 10.1111/evo.12593

[mec14523-bib-0039] Hardy, N. B. , & Otto, S. P. (2014). Specialization and generalization in the diversification of phytophagous insects: Tests of the musical chairs and oscillation hypotheses. Proceedings of the Royal Society of London B: Biological Sciences, 281, 20132960 https://doi.org/10.1098/rspb.2013.2960 10.1098/rspb.2013.2960 PMC4213601 25274368

[mec14523-bib-0040] Hawksworth, D. L. , & Luecking, R. (2017). Fungal diversity revisited: 2.2 to 3.8 million species. Microbiology spectrum, 5, 1–17, https://doi.org/10.1128/microbiolspec.FUNK-0052-2016. 10.1128/microbiolspec.funk-0052-2016 PMC11687528 28752818

[mec14523-bib-0041] Hedges, S. B. , Marin, J. , Suleski, M. , Paymer, M. , & Kumar, S. (2015). Tree of life reveals clock‐like speciation and diversification. Molecular biology and Evolution, 32, 835–845. https://doi.org/10.1093/molbev/msv037 25739733 10.1093/molbev/msv037 PMC4379413

[mec14523-bib-0042] Hohmann, N. , Wolf, E. M. , Lysak, M. A. , & Koch, M. A. (2015). A time‐calibrated road map of *Brassicaceae* species radiation and evolutionary history. The Plant Cell, 27, 2770–2784. 26410304 10.1105/tpc.15.00482 PMC4682323

[mec14523-bib-0043] Holst‐Jensen, A. , Kohn, L. , Jakobsen, K. , & Schumacher, T. (1997). Molecular phylogeny and evolution of *Monilinia* (*Sclerotiniaceae*) based on coding and noncoding rDNA sequences. American journal of botany, 84, 686 https://doi.org/10.2307/2445905 21708621

[mec14523-bib-0044] Holst‐Jensen, A. , Vaage, M. , & Schumacher, T. (1998). An approximation to the phylogeny of Sclerotinia and related genera. Nordic Journal of Botany, 18, 705–719. https://doi.org/10.1111/j.1756-1051.1998.tb01553.x

[mec14523-bib-0045] Holst‐Jensen, A. , Vrålstad, T. , & Schumacher, T. (2004). Kohninia linnaeicola, a new genus and species of the Sclerotiniaceae pathogenic to *Linnaea borealis* . Mycologia, 96, 135–142. https://doi.org/10.1080/15572536.2005.11833003 21148835

[mec14523-bib-0046] Hoshino, T. , Terami, F. , Tkachenko, O. B. , Tojo, M. , & Matsumoto, N. (2010). Mycelial growth of the snow mold fungus, *Sclerotinia borealis*, improved at low water potentials: An adaption to frozen environment. Mycoscience, 51, 98–103. https://doi.org/10.1007/S10267-009-0013-3

[mec14523-bib-0047] Hu, X. , Xiao, G. , Zheng, P. , Shang, Y. , Su, Y. , Zhang, X. , … Wang, C. (2014). Trajectory and genomic determinants of fungal‐pathogen speciation and host adaptation. Proceedings of the National Academy of Sciences of the United States of America, 111, 16796–16801. https://doi.org/10.1073/pnas.1412662111 25368161 10.1073/pnas.1412662111 PMC4250126

[mec14523-bib-0048] Janz, N. , & Nylin, S. (2008). The oscillation hypothesis of host‐plant range and speciation In: Tilmon K. (Ed.), Specialization, speciation, and radiation: The evolutionary biology of herbivorous insects (pp. 203–215). Berkeley, CA: Univ. of California Press.

[mec14523-bib-0049] Janz, N. , Nylin, S. , & Wahlberg, N. (2006). Diversity begets diversity: Host expansions and the diversification of plant‐feeding insects. BMC evolutionary biology, 6, 4 https://doi.org/10.1186/1471-2148-6-4 16420707 10.1186/1471-2148-6-4 PMC1382262

[mec14523-bib-0050] Johnson, K. P. , Malenke, J. R. , & Clayton, D. H. (2009). Competition promotes the evolution of host generalists in obligate parasites. Proceedings of the Royal Society of London B: Biological Sciences, 276, 3921–3926. https://doi.org/10.1098/rspb.2009.1174 10.1098/rspb.2009.1174 PMC2825781 19710056

[mec14523-bib-0051] Joshi, A. , & Thompson, J. N. (1995). Trade‐offs and the evolution of host specialization. Evolutionary Ecology, 9, 82–92. https://doi.org/10.1007/BF01237699

[mec14523-bib-0052] Katoh, K. , & Standley, D. M. (2013). mafft multiple sequence alignment software version 7: Improvements in performance and usability. Molecular biology and Evolution, 30, 772–780. https://doi.org/10.1093/molbev/mst010 23329690 10.1093/molbev/mst010 PMC3603318

[mec14523-bib-0053] Ketola, T. , Mikonranta, L. , Zhang, J. , Saarinen, K. , Ormälä, A. M. , Friman, V. P. , … Laakso, J. (2013). Fluctuating temperature leads to evolution of thermal generalism and preadaptation to novel environments. Evolution, 67, 2936–2944. 24094344 10.1111/evo.12148

[mec14523-bib-0054] Lefort, V. , Desper, R. , & Gascuel, O. (2015). FastME 2.0: A comprehensive, accurate, and fast distance‐based phylogeny inference program. Molecular biology and Evolution, 32, 2798–2800. https://doi.org/10.1093/molbev/msv150 26130081 10.1093/molbev/msv150 PMC4576710

[mec14523-bib-0055] Lewitus, E. , & Morlon, H. (2015). Characterizing and comparing phylogenies from their Laplacian spectrum. Systematic biology, 65, 495–507. 26658901 10.1093/sysbio/syv116

[mec14523-bib-0056] Liao, J. , Huang, H. , Meusnier, I. , Adreit, H. , Ducasse, A. , Bonnot, F. , … Morel, J. B. (2016). Pathogen effectors and plant immunity determine specialization of the blast fungus to rice subspecies. eLife, 5, e19377. 28008850 10.7554/eLife.19377 PMC5182064

[mec14523-bib-0057] Lorenzini, M. , & Zapparoli, G. (2016). Description of a taxonomically undefined Sclerotiniaceae strain from withered rotten‐grapes. Antonie van Leeuwenhoek, 109, 197–205. https://doi.org/10.1007/s10482-015-0621-1 26581438 10.1007/s10482-015-0621-1

[mec14523-bib-0058] Mbengue, M. , Navaud, O. , Peyraud, R. , Barascud, M. , Badet, T. , Vincent, R. , … Raffaele, S. (2016). Emerging trends in molecular interactions between plants and the broad host range fungal pathogens Botrytis cinerea and *Sclerotinia sclerotiorum* . Frontiers in plant science, 7, 422. 27066056 10.3389/fpls.2016.00422 PMC4814483

[mec14523-bib-0059] McManus, P. , Best, V. , & Voland, R. (1999). Infection of cranberry flowers by *Monilinia oxycocci* and evaluation of cultivars for resistance to cottonball. Phytopathology, 89, 1127–1130. https://doi.org/10.1094/PHYTO.1999.89.12.1127 18944635 10.1094/PHYTO.1999.89.12.1127

[mec14523-bib-0060] Melzer, M. , Smith, E. , & Boland, G. (1997). Index of plant hosts of *Sclerotinia minor* . Canadian Journal of Plant Pathology, 19, 272–280. https://doi.org/10.1080/07060669709500523

[mec14523-bib-0061] Merkle, D. , Middendorf, M. , & Wieseke, N. (2010). A parameter‐adaptive dynamic programming approach for inferring cophylogenies. BMC Bioinformatics, 11, S60 https://doi.org/10.1186/1471-2105-11-S1-S60 20122236 10.1186/1471-2105-11-S1-S60 PMC3009534

[mec14523-bib-0062] Moran, N. A. (1988). The evolution of host‐plant alternation in aphids: Evidence for specialization as a dead end. The American naturalist, 132, 681–706. https://doi.org/10.1086/284882

[mec14523-bib-0063] Morlon, H. , Lewitus, E. , Condamine, F. L. , Manceau, M. , Clavel, J. , & Drury, J. (2016). rpanda: An R package for macroevolutionary analyses on phylogenetic trees. Methods in Ecology and Evolution, 7(5), 589–597. https://doi.org/10.1111/2041-210X.12526

[mec14523-bib-0064] Nürk, N. M. , Uribe‐Convers, S. , Gehrke, B. , Tank, D. C. , & Blattner, F. R. (2015). Oligocene niche shift, Miocene diversification–cold tolerance and accelerated speciation rates in the St. John's Worts (Hypericum, Hypericaceae). BMC evolutionary biology, 15, 80 https://doi.org/10.1186/s12862-015-0359-4 25944090 10.1186/s12862-015-0359-4 PMC4422466

[mec14523-bib-0065] Nyman, T. , Linder, H. P. , Peña, C. , Malm, T. , & Wahlberg, N. (2012). Climate‐driven diversity dynamics in plants and plant‐feeding insects. Ecology letters, 15, 889–898. https://doi.org/10.1111/j.1461-0248.2012.01782.x 22507539 10.1111/j.1461-0248.2012.01782.x

[mec14523-bib-0066] Paradis, E. , Claude, J. , & Strimmer, K. (2004). APE: Analyses of phylogenetics and evolution in R language. Bioinformatics, 20, 289–290. https://doi.org/10.1093/bioinformatics/btg412 14734327 10.1093/bioinformatics/btg412

[mec14523-bib-0067] Poulin, R. , & Keeney, D. B. (2008). Host specificity under molecular and experimental scrutiny. Trends in parasitology, 24, 24–28. https://doi.org/10.1016/j.pt.2007.10.002 18023259 10.1016/j.pt.2007.10.002

[mec14523-bib-0068] Prieto, M. , & Wedin, M. (2013). Dating the diversification of the major lineages of Ascomycota (Fungi). PLoS One, 8, e65576 https://doi.org/10.1371/journal.pone.0065576 23799026 10.1371/journal.pone.0065576 PMC3683012

[mec14523-bib-0069] Qian, H. , & Zhang, J. (2014). Using an updated time‐calibrated family‐level phylogeny of seed plants to test for non‐random patterns of life forms across the phylogeny. Journal of systematics and evolution, 52, 423–430. https://doi.org/10.1111/jse.12086

[mec14523-bib-0070] Rabosky, D. L. (2014). Automatic detection of key innovations, rate shifts, and diversity‐dependence on phylogenetic trees. PLoS One, 9, e89543 https://doi.org/10.1371/journal.pone.0089543 24586858 10.1371/journal.pone.0089543 PMC3935878

[mec14523-bib-0071] Rabosky, D. L. , Grundler, M. , Anderson, C. , Shi, J. J. , Brown, J. W. , Huang, H. , & Larson, J. G. (2014). bammtools: An R package for the analysis of evolutionary dynamics on phylogenetic trees. Methods in Ecology and Evolution, 5, 701–707. https://doi.org/10.1111/2041-210X.12199

[mec14523-bib-0072] Rabosky, D. L. , Mitchell, J. S. , & Chang, J. (2017). Is BAMM flawed? Theoretical and practical concerns in the analysis of multi‐rate diversification models. Systematic biology, 66(4), 477–498. https://doi.org/10.1093/sysbio/syx037 28334223 10.1093/sysbio/syx037 PMC5790138

[mec14523-bib-0073] Rabosky, D. L. , Santini, F. , Eastman, J. , Smith, S. A. , Sidlauskas, B. , Chang, J. , & Alfaro, M. E. (2013). Rates of speciation and morphological evolution are correlated across the largest vertebrate radiation. Nature Communications, 4, 1958. 10.1038/ncomms2958 23739623

[mec14523-bib-0074] Razo‐Mendivil, U. , & De Leon, G. P.‐P. (2011). Testing the evolutionary and biogeographical history of Glypthelmins (Digenea: Plagiorchiida), a parasite of anurans, through a simultaneous analysis of molecular and morphological data. Molecular phylogenetics and evolution, 59, 331–341. https://doi.org/10.1016/j.ympev.2011.02.018 21356320 10.1016/j.ympev.2011.02.018

[mec14523-bib-0075] Revell, L. J. (2012). phytools: An R package for phylogenetic comparative biology (and other things). Methods in Ecology and Evolution, 3, 217–223. https://doi.org/10.1111/j.2041-210X.2011.00169.x

[mec14523-bib-0076] Ronquist, F. , Teslenko, M. , van der Mark, P. , Ayres, D. L. , Darling, A. , Höhna, S. , … Huelsenbeck, J. P. (2012). MrBayes 3.2: Efficient Bayesian phylogenetic inference and model choice across a large model space. Systematic biology, 61, 539–542. https://doi.org/10.1093/sysbio/sys029 22357727 10.1093/sysbio/sys029 PMC3329765

[mec14523-bib-0077] Sahoo, R. K. , Warren, A. D. , Collins, S. C. , & Kodandaramaiah, U. (2017). Hostplant change and paleoclimatic events explain diversification shifts in skipper butterflies (Family: Hesperiidae). BMC evolutionary biology, 17, 174 https://doi.org/10.1186/s12862-017-1016-x 28768477 10.1186/s12862-017-1016-x PMC5541431

[mec14523-bib-0078] Saito, I. (1997). Sclerotinia nivalis, sp. nov., the pathogen of snow mold of herbaceous dicots in northern Japan. Mycoscience, 38, 227 https://doi.org/10.1007/BF02460857

[mec14523-bib-0079] Schliep, K. P. (2011). phangorn: Phylogenetic analysis in R. Bioinformatics, 27(4), 592–593. https://doi.org/10.1093/bioinformatics/btq706 21169378 10.1093/bioinformatics/btq706 PMC3035803

[mec14523-bib-0080] Schoch, C. L. , Seifert, K. A. , Huhndorf, S. , Robert, V. , Spouge, J. L. , Levesque, C. A. , … Fungal Barcoding Consortium (2012). Nuclear ribosomal internal transcribed spacer (ITS) region as a universal DNA barcode marker for Fungi. Proceedings of the National Academy of Sciences of the United States of America, 109, 6241–6246. https://doi.org/10.1073/pnas.1117018109 22454494 10.1073/pnas.1117018109 PMC3341068

[mec14523-bib-0081] Schumacher, T. , & Kohn, L. M. (1985). A monographic revision of the genus *Myriosclerotinia* . Canadian Journal of Botany, 63, 1610–1640. https://doi.org/10.1139/b85-224

[mec14523-bib-0082] Smith, S. A. , Beaulieu, J. M. , & Donoghue, M. J. (2010). An uncorrelated relaxed‐clock analysis suggests an earlier origin for flowering plants. Proceedings of the National Academy of Sciences of the United States of America, 107, 5897–5902. https://doi.org/10.1073/pnas.1001225107 20304790 10.1073/pnas.1001225107 PMC2851901

[mec14523-bib-0083] Spanu, P. D. , Abbott, J. C. , Amselem, J. , Burgis, T. A. , Soanes, D. M. , Stüber, K. , … Panstruga, R. (2010). Genome expansion and gene loss in powdery mildew fungi reveal tradeoffs in extreme parasitism. Science, 330, 1543 https://doi.org/10.1126/science.1194573 21148392 10.1126/science.1194573

[mec14523-bib-0084] Staats, M. , Van Baarlen, P. , & Van Kan, J. A. (2005). Molecular phylogeny of the plant pathogenic genus *Botrytis* and the evolution of host specificity. Molecular biology and Evolution, 22, 333–346. 15496556 10.1093/molbev/msi020

[mec14523-bib-0085] Strömberg, C. A. (2011). Evolution of grasses and grassland ecosystems. Annual Review of Earth and Planetary Sciences, 39, 517–544. https://doi.org/10.1146/annurev-earth-040809-152402

[mec14523-bib-0086] Thines, M. , & Choi, Y.‐J. (2015). Evolution, diversity, and taxonomy of the Peronosporaceae, with focus on the genus Peronospora. Phytopathology, 106, 6–18. 26649784 10.1094/PHYTO-05-15-0127-RVW

[mec14523-bib-0087] Vander Wal, E. , Garant, D. , Calmé, S. , Chapman, C. A. , Festa‐Bianchet, M. , Millien, V. , … Pelletier, F. (2014). Applying evolutionary concepts to wildlife disease ecology and management. Evolutionary applications, 7, 856–868. https://doi.org/10.1111/eva.12168 25469163 10.1111/eva.12168 PMC4227862

[mec14523-bib-0088] Visser, B. , Le Lann, C. , Den Blanken, F. J. , Harvey, J. A. , Van Alphen, J. J. , & Ellers, J. (2010). Loss of lipid synthesis as an evolutionary consequence of a parasitic lifestyle. Proceedings of the National Academy of Sciences of the United States of America, 107, 8677–8682. https://doi.org/10.1073/pnas.1001744107 20421492 10.1073/pnas.1001744107 PMC2889307

[mec14523-bib-0089] Walker, A. S. , Gautier, A. L. , Confais, J. , Martinho, D. , Viaud, M. , Le Pêcheur, P. , … Fournier, E. (2011). Botrytis pseudocinerea, a new cryptic species causing gray mold in French vineyards in sympatry with *Botrytis cinerea* . Phytopathology, 101, 1433–1445. https://doi.org/10.1094/PHYTO-04-11-0104 21830954 10.1094/PHYTO-04-11-0104

[mec14523-bib-0090] Woolhouse, M. E. , & Gowtage‐Sequeria, S. (2005). Host range and emerging and reemerging pathogens. Emerging Infectious Diseases, 11, 1842–1847. https://doi.org/10.3201/eid1112.050997 16485468 10.3201/eid1112.050997 PMC3367654

[mec14523-bib-0091] Woolhouse, M. E. , Taylor, L. H. , & Haydon, D. T. (2001). Population biology of multihost pathogens. Science, 292, 1109–1112. https://doi.org/10.1126/science.1059026 11352066 10.1126/science.1059026

[mec14523-bib-0092] Yang, Z. , Kumar, S. , & Nei, M. (1995). A new method of inference of ancestral nucleotide and amino acid sequences. Genetics, 141, 1641–1650. 8601501 10.1093/genetics/141.4.1641 PMC1206894

[mec14523-bib-0093] Yu, Y. , Harris, A. J. , Blair, C. , & He, X. (2015). RASP (Reconstruct Ancestral State in Phylogenies): A tool for historical biogeography. Molecular phylogenetics and evolution, 87, 46–49. https://doi.org/10.1016/j.ympev.2015.03.008 25819445 10.1016/j.ympev.2015.03.008

[mec14523-bib-0094] Zachos, J. , Pagani, M. , Sloan, L. , Thomas, E. , & Billups, K. (2001). Trends, rhythms, and aberrations in global climate 65 Ma to present. Science, 292, 686–693. https://doi.org/10.1126/science.1059412 11326091 10.1126/science.1059412

