# Supplementary material for: Shifts in diversification rates and host jump frequencies shaped the diversity of host range among Sclerotiniaceae fungal plant pathogens
Source: Mol Ecol. 2018 Mar 23;27(5):1309–23. doi: 10.1111/mec.14523 (PMC5900718; doi:10.1111/mec.14523)
Supplement: Supplementary file 1 [file MEC-27-1309-s001.zip › Supplementary_files_readme.pdf]

Shifts in diversification rates and host jump frequencies shaped the diversity of host range among *Sclerotiniaceae* fungal plant pathogens

Olivier Navaud, Adelin Barbacci, Andrew Taylor, John P. Clarkson, Sylvain Raffaele

SUPPLEMENTARY FILES

Index of files available in the .zip archive:

Supplementary File 1. Phylogenetic tree of host plant families used for co-phylogenetic analyses in this work (newick format).

Supplementary File 2. Curated multiple ITS sequence alignment for 200 Leotiomycete species, including 105 *Sclerotiniaceae* and 56 *Rutstroemiaceae* species. This alignment includes 797 informative sites and was used to generate the phylogenetic tree shown in Figure 1 and downstream analyses (fasta format).

Supplementary File 3. Phylogenetic tree of 200 Leotiomycete species including 161 *Sclerotiniaceae* and *Rutstroemiaceae* species used in Figure 1, obtained by maximum likelihood approach and featuring SH-aLRT branch support (newick format).

Supplementary File 4. Same phylogenetic tree as in supplementary file 3 including bootstrap from 100 replicates as branch support (newick format).

Supplementary File 5. Time calibrated phylogenetic tree of the 105 *Sclerotiniaceae* species used in Figure 2.

Supplementary File 6. List of host-parasite associations tested for co-phylogenetic analyses.

Supplementary Table 1. List of *Sclerotiniaceae* and *Rutstroemiaceae* species used for phylogenetic analysis and their corresponding host range. 1 refers to position in the tree shown in Figure 1; 2 refers to the code used in RASP analysis (Sup. Figure 5). NA, not applicable; Rutst., *Rutstroemiaceae*; Sclero. *Sclerotiniaceae*.

These files are also available from the Dryad Digital Repository: https://doi.org/10.5061/dryad.7cs3g
