# Supplementary material for: Shifts in diversification rates and host jump frequencies shaped the diversity of host range among Sclerotiniaceae fungal plant pathogens
Source: Mol Ecol. 2018 Mar 23;27(5):1309–23. doi: 10.1111/mec.14523 (PMC5900718; doi:10.1111/mec.14523)
Supplement: Supplementary file 2 [file MEC-27-1309-s002.pdf]

Shifts in diversification rates and host jump frequencies shaped the diversity of host range among *Sclerotiniaceae* fungal plant pathogens

Olivier Navaud, Adelin Barbacci, Andrew Taylor, John P. Clarkson, Sylvain Raffaele

SUPPLEMENTARY FIGURES

Supplementary Figure 1. We analyzed the 7101 records from the fungus-host distribution database that served as a basis for our study on the *Sclerotiniaceae*. Host species were classified as crops according to the USDA Natural Resources Conservation Service database (https://plants.usda.gov/npk/main), other cultivated plants, ornamental plants and "others", including largely wild plant species. We found that 13% of the Fungus-Host database entries related to crops, 20% to cultivated plants and 9.5% to ornamentals (A). A total of 37% of the Fungus-Host database entries were from the USA, 17% from Europe, leaving a total of 46% of the entries that were neither from the USA or Europe (B). These distributions do not reveal a strong bias towards crops or USA/Europe records in the records used for this analysis. (C) Relationship between fungal pathogen host range and the number of records in the database for each plant family. Dotted line shows linear regression of the data.

Supplementary Figure 2. Reconstruction of ancestral host range by the re-rooting method under an entity-relationship model and robustness of the results to tree pruning. (A) Posterior probability for the presence of Fabids in *Sclerotiniaceae* at each internal node (red), Fabids being the most likely ancestral host group of the *Sclerotiniaceae* family in S-DIVA and S-DEC analyses. (B) Sensitivity to tree pruning for the date of inclusion of plant groups into *Sclerotiniaceae* pathogen host range. The graph shows the % of variation for the earliest date of inclusion into *Sclerotiniaceae* pathogen host range upon pruning of 10% of the phylogenetic tree 100 times randomly. Error bars show 95% confidence intervals. (C) Posterior probability for the presence of *Rosaceae* (a family in the Fabids) in *Sclerotiniaceae* host groups at each internal node (red).

Supplementary Figure 3. Assessment of the robustness of diversification rate shifts predicted by MEDUSA to sampling richness (A-B), tree completeness (C-D) and dating accuracy (E-F). The figure shows the frequency of detection of a diversification rate shift at every internal node of the tree in 100

bootstrap MEDUSA analyses (A, C and E), as well as the total number of shifts predicted in each of the 100 bootstrap analyses (B, D and F).

Supplementary Figure 4. Diversification rates analysis with R-PANDA. (A) Spectral density plot of the *Sclerotiniaceae*. (B) Eigenvalues ranked in descending order for the *Sclerotiniaceae*, the eigengap between values 8 and 9 is shown by an arrow and indicates eight modalities in this phylogeny. (C) Location of the eight modalities on the *Sclerotiniaceae* phylogeny, indicated by different colors. The major rate shifts identified by BAMM are shown as circles on the phylogeny and delimit the shift between modes 1/7/8 to modes 5/6/3 and finally modes 2/4.

Supplementary figure 5. RASP phylogram showing extant and reconstructed ancestral host range for 161 *Sclerotiniaceae* and *Rutstroemiaceae* species. The ancestral reconstruction shows the probabilities associated with the optimal distribution at major nodes. It was generated using the S-DIVA method and the phylogenetic tree provided in supplementary file 2 as a template. The code for host groups is as follows: Vitales (A), Asterids (B), Campanuliids (C), Commelinids (D), coprophilous (E), core Eudicots (F), Eudicots (G), Fabids (H), Polypodiidae (I), Lamiids (J), Magnoloidae (K), Malvids (L), Monocotyledones (M), Pinidae (N).

Supplementary Figure 6. Chronotree of the 105 *Sclerotiniaceae* species generated with BEAST. Estimated mean age of all intermediate nodes is indicated (in million years), with bars showing the 95% confidence interval of the highest posterior density (HPD).

Supplementary Figure 7. Diversification rates analysis with BAMM and Medusa. (A) The eight most probable shift configurations in the 95% credible set of shift configurations predicted by BAMM with the posterior probability of each configuration indicated on top. (B) Density distribution of net diversification rates calculated by BAMM. The three peaks of density reflect three macro-evolutionary regimes in the *Sclerotiniaceae*. (C) Net diversification rates as a function of time calculated by BAMM through the complete *Sclerotiniaceae* phylogeny (105 species), showing a first a relatively sharp decrease (70 to 55 Mya), followed by a slow decrease (55 to 17 Mya) and a recent increase (17 Mya to present) in diversification rates. (D) Diversification rate shifts identified by MEDUSA.
